# Non-destructive quantification of key quality characteristics in individual grapevine berries using near-infrared spectroscopy

**DOI:** 10.3389/fpls.2024.1386951

**Published:** 2024-07-05

**Authors:** Lucie Cornehl, Pascal Gauweiler, Xiaorong Zheng, Julius Krause, Florian Schwander, Reinhard Töpfer, Robin Gruna, Anna Kicherer

**Affiliations:** ^1^ Institute for Grapevine Breeding Geilweilerhof, Julius Kühn-Institute, Federal Research Centre of Cultivated Plants, Siebeldingen, Germany; ^2^ Fraunhofer Institute of Optronics, System Technologies and Image Exploitation IOSB, Karlsruhe, Germany; ^3^ Vision and Fusion Laboratory (IES), Department of Informatics, Karlsruhe Institute of Technology, Karlsruhe, Germany

**Keywords:** maturity, NIRS, precision viticulture, quality, handheld, field phenotyping, phenomics non-destructive quality quantification using NIRS

## Abstract

It is crucial for winegrowers to make informed decisions about the optimum time to harvest the grapes to ensure the production of premium wines. Global warming contributes to decreasing acidity and increasing sugar levels in grapes, resulting in bland wines with high contents of alcohol. Predicting quality in viticulture is thus pivotal. To assess the average ripeness, typically a sample of one hundred berries representative for the entire vineyard is collected. However, this process, along with the subsequent detailed must analysis, is time consuming and expensive. This study focusses on predicting essential quality parameters like sugar and acid content in *Vitis vinifera* (L.) varieties ‘Chardonnay’, ‘Riesling’, ‘Dornfelder’, and ‘Pinot Noir’. A small near-infrared spectrometer was used measuring non-destructively in the wavelength range from 1 100 nm to 1 350 nm while the reference contents were measured using high-performance liquid chromatography. Chemometric models were developed employing partial least squares regression and using spectra of all four grapevine varieties, spectra gained from berries of the same colour, or from the individual varieties. The models exhibited high accuracy in predicting main quality-determining parameters in independent test sets. On average, the model regression coefficients exceeded 93% for the sugars fructose and glucose, 86% for malic acid, and 73% for tartaric acid. Using these models, prediction accuracies revealed the ability to forecast individual sugar contents within an range of ± 6.97 g/L to ± 10.08 g/L, and malic acid within ± 2.01 g/L to ± 3.69 g/L. This approach indicates the potential to develop robust models by incorporating spectra from diverse grape varieties and berries of different colours. Such insight is crucial for the potential widespread adoption of a handheld near-infrared sensor, possibly integrated into devices used in everyday life, like smartphones. A server-side and cloud-based solution for pre-processing and modelling could thus avoid pitfalls of using near-infrared sensors on unknown varieties and in diverse wine-producing regions.

## 1 Introduction

Due to the influence of climate change, the wine industry faces significant challenges in maintaining the production of high-quality wines (Rogiers et al., 2022). For Europe phenology, i.e. flowering, véraison and harvest dates, are predicted to take place earlier in the year (Duchêne et al., 2010), which is supported by a long term experiment in Alsace, France (Delrot et al., 2020). Consequently, grapes ripen earlier and under higher temperatures leading to elevated sugar levels and reduced acidity in the grapes, that result in wines with higher alcohol content and a less pronounced acidity. Additionally, high sugar levels are achieved prior to phenolic maturity, impacting the aroma profile of wines. Viticulturists are compelled to adapt to those changes and their subsequent effects. Apart from the cultivation of new grape varieties with improved resistance, endeavors are underway to breed cultivars with a delayed onset of ripening (Duchêne et al., 2010) and to explore and adopt viticultural practices that prove advantageous in anticipated conditions (Santos et al., 2020). Moreover, there is increasing recognition of the significance of phenotyping, as it can aid both practical decision-making and scientific applications.

Grapevine is a perennial crop that has a significant role in promoting healthy nutrition and is of cultural and economic importance for wine production. After the dormant period and the subsequent emergence of new branches, leaves, and inflorescences, the process of berry formation and ripening commences. The ripening course follows a double sigmoid pattern and can be divided into three phases (Coombe, 1992). In the first growing phase acids are produced. This is followed by a lag phase, where berry growth ceases. The end of this period and the onset of the second growing phase coincide with berry colouring and softening. This is known as véraison during which the berries begin to accumulate sugars (Conde et al., 2007). The second growing phase represents the maturation stage, characterised by significant sugar accumulation and a decrease in acid concentration, either through dilution or metabolic processes (Ollat et al., 2002). High temperatures during this phase are believed to drive the reduction in acid content and the storage of sugars (Duchêne et al., 2010; Bock et al., 2011; Urhausen et al., 2011). This underscores the importance for winegrowers to determine the optimal harvest date in order to minimise economic damage.

Upon reaching technological ripeness, characterised by sweet berries with sufficient acidity, the harvest should commence. The determination of this time can be gauged through the average ripeness of the entire vineyard. To evaluate this by measuring the concentrations of the quality-determining substances, winegrowers collect a sample comprising one hundred berries. Important quality traits are measured, among others things, contents of sugars, acids and total phenols. Because of methods like Fourier-transform infrared spectroscopy (FTIR) or high-performance liquid chromatography (HPLC) are confined to the laboratory, they may not always be utilised to ascertain the best date of harvesting. The interest in predicting the ripeness, quality or durability of fruit and vegetable using Near-infrared Spectroscopy (NIRS) has been a subject of research for some time now (Huang et al., 2008; Goisser et al., 2019; Kalopesa et al., 2023) and feasibility for the use in viticulture was proven multiple times (Cozzolino et al., 2006). The spectra were captured using a range of costly (González-Caballero et al., 2012) spectrometers for measurements in reflectance mode (Xiao et al., 2018), with some of them now being unavailable.

It can be noticed that a more distinct trend towards miniaturisation is evident (Grüger et al., 2018; Rodriguez-Saona et al., 2020; Grüger et al., 2023), as this forms the basis for increased portability, user-friendliness and a broader applicability. Moreover, miniaturised sensors, whether integrated into or externally connected to mobile phones, are likely to be considerably more cost-effective (Das et al., 2016). Therefore, the trend is currently leaning towards a smartphone-based sensor system, which has already been presented in the end of 2023 (trinamiX GmbH, Ludwigshafen, Germany). However, despite technological progress and the successful evaluation of chemometric models, widespread use of this technique in viticulture requires already existing robust models, an easy to operate calibration of the instruments or, the most user-friendly method, a database for cloud-based processing and the respective modelling. Another upcoming challenge in using near-infrared sensors is the transferability of calibrations to other sensors. Especially when they are integrated into smartphones, which undergo rapid technological development, calibration models should be transferable to other sensors and robust against temperature changes, as investigated by Mishra et al. (2020). Robust models require big data sets with high quality spectra and precise reference values for the calibration process (Walsh et al., 2020). These must be collected over the entire ripening processes to encompass the widest possibly range of values. This is essential for establishing dependable models and accurately determining the components, particularly in ripe grapes. Additionally, variances in spectra could be observed for different varieties and several studies stated that there is also a geographic influence (Arana et al., 2005; Liu et al., 2006b; Cozzolino et al., 2011; Martelo-Vidal et al., 2013; Zheng et al., 2020). This presents challenges due to the multitude of prominent wine-producing countries across Europe. (Food and Agriculture Organization of the United Nations, 2023) Moreover, in Germany alone, about 300 different grapevine varieties are permitted to be cultivated and refined for the production of wine (Bundesanstalt für Landwirtschaft und Ernährung, 2023). While sensors are becoming more affordable and smaller, making their way into our everyday lives, calibrating the parameters would consequently skyrocket the cost of such a solution. This is due to the multitude of factors to consider and the vast datasets involved. Therefore, ways should be explored to approximately predict the relevant constituents of grape berries from previously uncalibrated varieties and regions. Additionally, enabling continuous calibration through the use by winemakers and hobby oenologists via cloud solutions should be considered.

In this study a large data set was created, enabling an automatic calculation of different models for the prediction of four quality-determining substances. Models were specifically developed using berries of all four varieties, from both white and red berries, and from the berries of individual varieties, and were then tested. The ability of forecasting the contents of the sugars fructose and glucose, as well as the acids malic acid and tartaric acid are then compared. An independent data set of ripe berries from four economically important *Vitis vinifera* (L.) varieties was created and the resulting prediction accuracies for ripeness stages expected by winegrowers were compared.

## 2 Material and methods

### 2.1 Plant material

Red (‘Dornfelder’, ‘Pinot Noir’) and white (‘Chardonnay’, ‘Riesling’) *Vitis vinifera* (L.) varieties were chosen due to their significance in German wine market. ‘Pinot Noir’ and ‘Riesling’, the two most significant grape varieties, jointly occupy over 69% of Germany’s vineyard area. Combined, the four varieties contribute to over 94% of the wine grape areas in Germany (Deutsches Weininstitut GmbH, 2022). Additionally they also serve as typical examples of red and white vines that play a crucial role in the production of high-quality wines.

**Figure 1:**
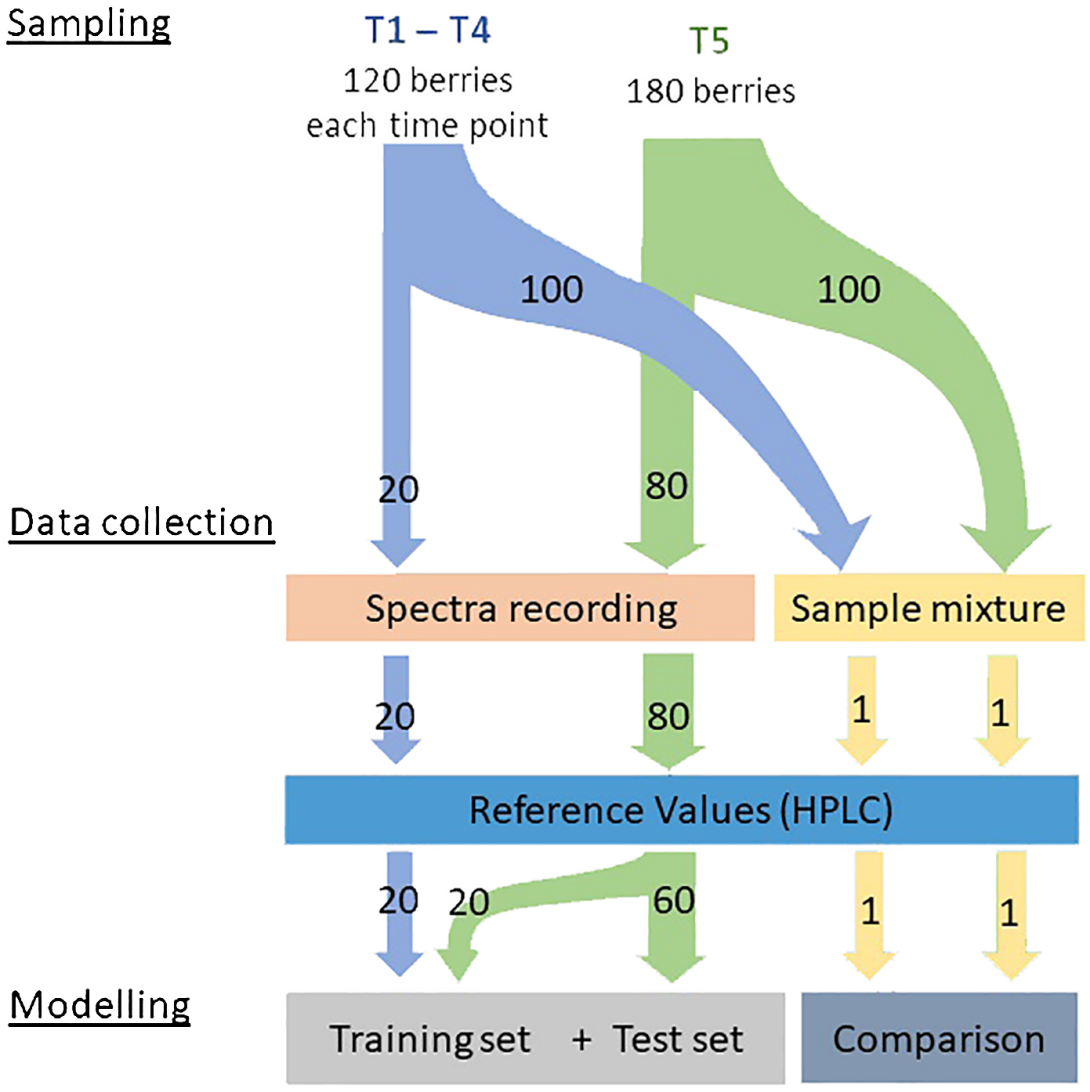
Scheme for the sampling of individual grapevine berries, spectra and acquisition of corresponding reference values.

Samples were taken from four different commercial vineyards located in the vineyard site “Wollmesheimer Mütterle”, next to the town Landau in the south of Rhineland Palatinate, Germany (49°10’41.8”N 8°05’36.7”E). Red (‘Dornfelder’, ‘Pinot Noir’) and white (‘Chardonnay’, ‘Riesling’) *Vitis vinifera* (L.) varieties grew on these plots. All vineyards were north-south oriented and well-tended. Sampling took place onwards from véraison, from 1^st^ of August 2022 till 26^th^ of September 2022 on five sampling dates per variety (see **Table 1**). On each day 120 (first four sampling dates) or 180 individual berries (last sampling date) were collected randomly, but evenly distributed over the entire plot. From these individual berries, one hundred berries were used for a mixed sample, as a reference for the average ripeness of the plots. The remaining berries were used for the collection of spectra and single berry reference values as described in the following paragraphs (see **Figure 1**).

**Table 1:** Information on the vineyards of the used *Vitis vinifera* (L.) varieties.

*Vitis vinifera* (L.) Variety	Year of planting	Farming practice	Root-stock	Date of sampling				
				T1	T2	T3	T4	T5
‘Chardonnay’	2001	Org	SO4	08–03	08–11	08–23	09–05	09–12
‘Riesling’	1991	Org	Binova	08–04	08–18	08–25	09–14	09–26
‘Dornfelder’	2002	Con	125AA	08–01	08–10	08–19	09–07	09–21
‘Pinot Noir’	1990	Org	SO4	08–02	08–15	08–24	09–06	09–17

Shown are the corresponding years of planting, the applied farming practices with either organic (Org) or conventional (Con) management, the rootstocks on which the varieties are grafted and the dates of taking samples (T1–T5) in the year 2022.

### 2.2 Collection of spectral data

Spectra of 20 (T 1–4) and 80 (T 5) individual berries, respectively, were recorded at the same day as sampling took place in the laboratory with the NIRONE Sensor S 1.4 (SpectralEngines, Steinbach, Germany). The sensor covered the wavelength range from 1 100 nm to 1 350 nm (512 wavelength bands) and was used in reflectance mode using the designated included front optic. The sensor has a resolution of 12 nm - 16 nm, uses a single element InGaAs detector and a Fabry-Pérot interferometer for optical filtering. Signal-to-noise ratio of the sensor is 15 000. Berries were illuminated by the two integrated tungsten vacuum lamps. Measurements were conducted using the Fraunhofer SmartSpectrometer System (Krause et al., 2021). Spectra collection took place in a room with a consistent temperature and without artificial ceiling lighting. Prior to placing the clean and unfogged berry on the cleansed surface of the sensor, white and black calibration was performed using a 99% Spectralon diffuse reflection standard (Sphereoptics, Labsphere, Inc., North Sutton, NH, USA) with a diameter of 2.54 cm. For each individual berry three spectra from different sites of the berry were recorded and averaged to represent the berry as good as possible. Each wavelength was recorded 100 times within the sensor (measuring time 1.536 s) and averaged. This improved the signal-to-noise ratio by a factor of 10.

### 2.3 Sample processing and acquisition of reference values

After the recording of spectral data, reference values were determined using high-performance liquid-chromatography (HPLC). To gain the must, individual berries were put in a 50ml Falcon tube with four stainless steel beads (diameter 5.00mm). Berries were destroyed by shaking in a paint shaker (SK450, Fast and Fluid Management, Sassenheim, Netherlands). After removing the beads, the Falcon tubes were centrifuged at 25 418 · g (Sigma 6–16ks, Sigma Laborzentrifugen GmbH, Osterode am Harz, Germany) for 6min and the clear supernatant was decanted in a 2ml tube. The remaining one hundred berries were crushed with a blender (BL6280, Grundig, Germany). Must was centrifuged at 20340 · g (Sigma 6K15, Sigma Laborzentrifugen GmbH, Osterode am Harz, Germany) for 10min and poured through a 100µm sieve into Falcon tubes. A subsample was transferred into a 2ml tube. The must of the individual berries and the subsamples of must from the one hundred berries samples were again centrifuged at 12100 · g (Minispin Eppendorf, Hamburg, Germany) for 6min and 1:3 diluted with double distilled, filtered (pore size 0.2nm) water. After mixing and another centrifugation step, 150µl were filled into vials. Multi-component standard four stage dilution series was created containing 1.5g/L to 90g/L fructose and glucose, and 0.15g/L to 9g/l malic acid and tartaric acid as well. The vials containing the 1:3 diluted samples and the respective multi-component standards were placed in the multisampler (G7167B) for subsequent HPLC (Agilent 12900 Infinity II, Agilent Technologies Inc., Santa Clara, CA, USA) analysis. The system was equipped with a binary pump (G7120A) and a column oven (G7116B) kept at 75°C for separation. The Rezex ROA-Organic AcidH^+^ ion exclusion column (300mm × 7.8mm, 8µm) was protected by a security guard Carbo-H^+^ column (Phenomenex Inc., Torrance, CA, USA). 5µl of the samples were injected and analysed in a 16.5min run under an aqueous mobile phase of 0.4mM sulphuric acid and at a flow rate of 0.06mL/min. Malic acid and tartaric acid were detected using a diode array detector (G7117B) at 210.4nm and the sugars fructose and glucose with a refractive index detector (G1362A) kept at 50°C. As an internal control after every up to ten samples one stage of the dilution series was injected. The correct identification of the peaks was checked visually and adjusted if necessary. Subsequent data analysis was performed using Agilent OpenLab Chemstation software (Agilent Technologies Inc., Santa Clara, CA, USA).

### 2.4 Modelling

During modelling, the data were split into a set for training and optimisation and a set for testing. The training set was defined as all spectra at time T 1 to T 4 and 20 spectra at time T 5. This resulted in 100 spectra for each variety, comprising 20 spectra from each time point (see **Figure 1**). The remaining 60 spectra at time T 5 were defined as the independent test set. To prevent a random split, which can lead to an unfavourable division in the sense of a poor representation of the data, the Kennard Stone Algorithm (Kennard and Stone, 1969) was applied. This approach helped to include spectra with the highest reference values in the training set, to prevent skewed prediction results due to extrapolation.

To calculate robust models, pre-processing of the spectra is essential. In accordance with Schorn-García et al. (2023) the following two pre-processing techniques work best. The spectra were smoothed using the Savitzky-Golay algorithm (SG) (Savitzky and Golay, 1964), to mitigate noise. This algorithm replicates the spectrum with a polynomial of a specified order within a defined window size. The polynomial value becomes the new spectrum value at that point, iterating over each point of the spectrum. Moreover, derivative methods provided by the algorithm were utilised to eliminate baseline effects up to the respective order. Additionally, all spectra underwent normalisation using the standard normal variate (SNV) method (Barnes et al., 1989). This involves subtracting the mean spectrum value and dividing it by its standard deviation.

Using the training set, seven models were created for each content. One that contains the data of all four varieties, one that contains the data of the white (‘Chardonnay’, ‘Riesling’) and one that contains the data of the red (‘Dornfelder’, ‘Pinot Noir’) varieties and finally for each variety individually. Consequently, the number of spectra included for modelling varies from 398 to 99 due to the different scopes of the individual models (see **Table 2**). For the modelling, a partial least square regression (PLSR) (Wold et al., 2001) was used, which is a standard method in chemometric data analysis. The PLSR has one degree of freedom: the number of components. Selecting too few components results in a model that is too general and yields poor predictions. Conversely, choosing too many components leads to overfitting and a lack of adaptability to new data. To address this additional rules were introduced into the automatic optimisation process, as described below.

**Table 2:** Distribution of the data used for modelling.

Model	Spectra	*Training Set*				*Test Set*			
		CHA	RIE	DOR	PIN	CHA	RIE	DOR	PIN
All:									
All *Colour:*	(n=398)	99	99	100	100	60	60	60	60
White	(n=198)	99	99	–	–	60	60	–	–
Red *Individual:*	(n=200)	–	–	100	100	–	–	60	60
CHA	(n=99)	99	–	–	–	60	–	–	–
RIE	(n=99)	–	99	–	–	–	60	–	–
DOR	(n=100)	–	–	100	–	–	–	60	–
PIN	(n=100)	–	–	–	100	–	–	–	60

Training set contains all spectra at time T1 to T4 and 20 spectra at time T5. Test set contains 60 spectra from T5 (see **Table 1**) for each *Vitis vinifera* (L.) variety comprising ‘Chardonnay’ (CHA), ‘Riesling’ (RIE), ‘Dornfelder’ (DOR) and ‘Pinot Noir’ (PIN). The Kennard Stone algorithm was used for the representative distribution of the test data at time T5. This distribution was used for each content of interest.

Alltogether, there are a total of four parameters for pre-processing and regression. These are the size of the window, the polyorder and the degree of derivation in Savitzky-Golay algorithm and the number of components in PLSR. Each of these parameters has an impact on the prediction accuracy. To find the best combination, a brute-force approach was used, which creates models with all possible combinations and outputs the parameters of the best performing model. To validate the training results, a cross validation with five partitions was used. The best combination was defined as the one with the lowest root mean square error (RMSE) of the validation set (CV-RMSE). For computing reasons, the parameters are limited to the range as follows: window size: 5–41, polyorder: 1–4, degree of derivation: 0–2, number of components: 1–12. To prevent overfitting, an additional rule was introduced. The CV-RMSE must be lower than 97% of the combination with the currently best parameters. Using the best combination of the pre-processing parameters and the number of components, the final model was created with all data from the training set after the calculated pre-processing. Finally, the test set was also pre-processed regarding to the optimisation results and passed to the model. The parameters of the respective model are listed in **Table 3**.

**Table 3:** Parameters of the respective models and target substance.

Model:	All	Colour			Individual		
Analyte	All	White	Red	CHA	RIE	DOR	PIN
Fructose							
win.s.	5	5	5	5	5	19	7
poly.	1	1	1	1	1	1	1
deriv.	0	0	0	0	0	0	0
PLSR comp.	3	3	3	3	3	5	7
*Glucose*							
win.s.	5	39	5	5	5	31	5
poly.	1	1	1	2	1	1	1
deriv.	0	0	0	0	0	0	0
PLSR comp.	3	11	3	10	3	6	3
*Malic acid*							
win.s.	29	21	25	25	25	25	37
poly.	1	1	1	1	1	1	1
deriv.	0	0	0	0	0	0	0
PLSR comp.	6	8	5	7	6	9	5
*Tartaric acid*							
win.s.	21	5	31	5	5	29	5
poly.	1	1	1	1	1	1	1
deriv.	0	0	0	0	0	0	0
PLSR comp.	5	3	5	3	3	4	3

Savitzky-Golay’s window size (win.s.), polyorder (poly.) and derivative (deriv.) and number of components of PLSR (PLSR comp.).

**Figure 2:**
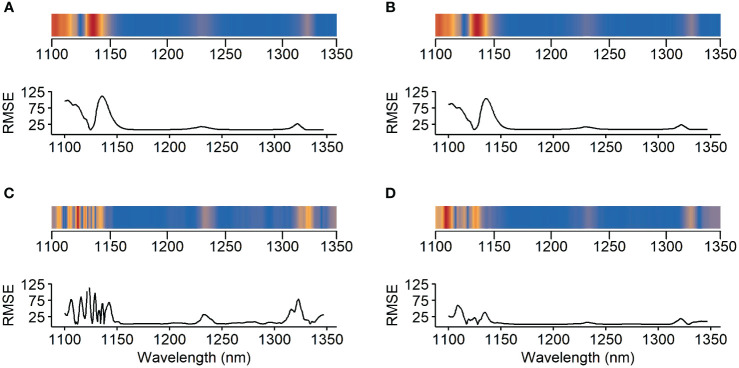
Attribution map of the different models for the target substances **(A)** fructose, **(B)** glucose, **(C)** malic acid and **(D)** tartaric acid. Parts of the spectrum were masked out with a window width of 5. The resulting root mean square error (RMSE) of each point is an indicator of the significance of this wavelength range. The higher the RMSE, the more important the wavelength (red areas).

Additionally, an attribution map was created (**Figure 2**) to find out which wavelength is decisive for predicting. Parts of the spectrum were masked out with a window width of 5, i.e. set to 0, and thus fed into the model. This window iterates over every spectral point. The resulting RMSE of each point is an indicator of the significance of this wavelength range. A higher RMSE means that this spectral range is more important for the prediction.

## 3 Results

### 3.1 Spectral data set

**Figure 3:**
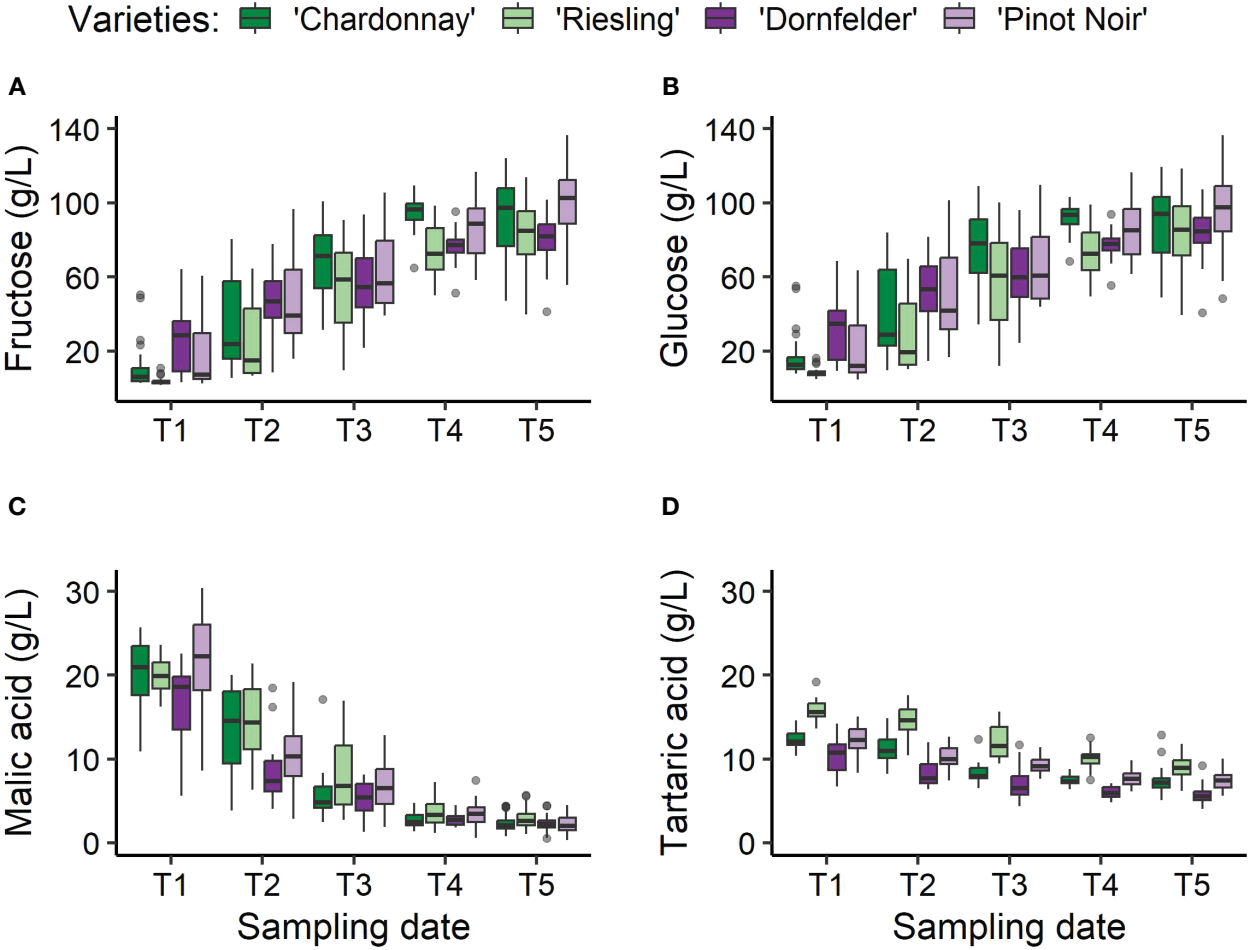
Changes in the key quality-defining ingredients within the grapevine berries of *Vitis vinifera* (L.) varieties ‘Chardonnay’ (dark green), ‘Riesling’ (light green), ‘Dornfelder’ (dark purple), and ‘Pinot Noir’ (light purple), over the sampling period from T1 to T5 (refer to **Table 1**). Contents of the sugars fructose **(A)** and glucose **(B)**, and of the acids malic acid **(C)** and tartaric acids **(D)** were determined as a reference using HPLC.

**Figure 4:**
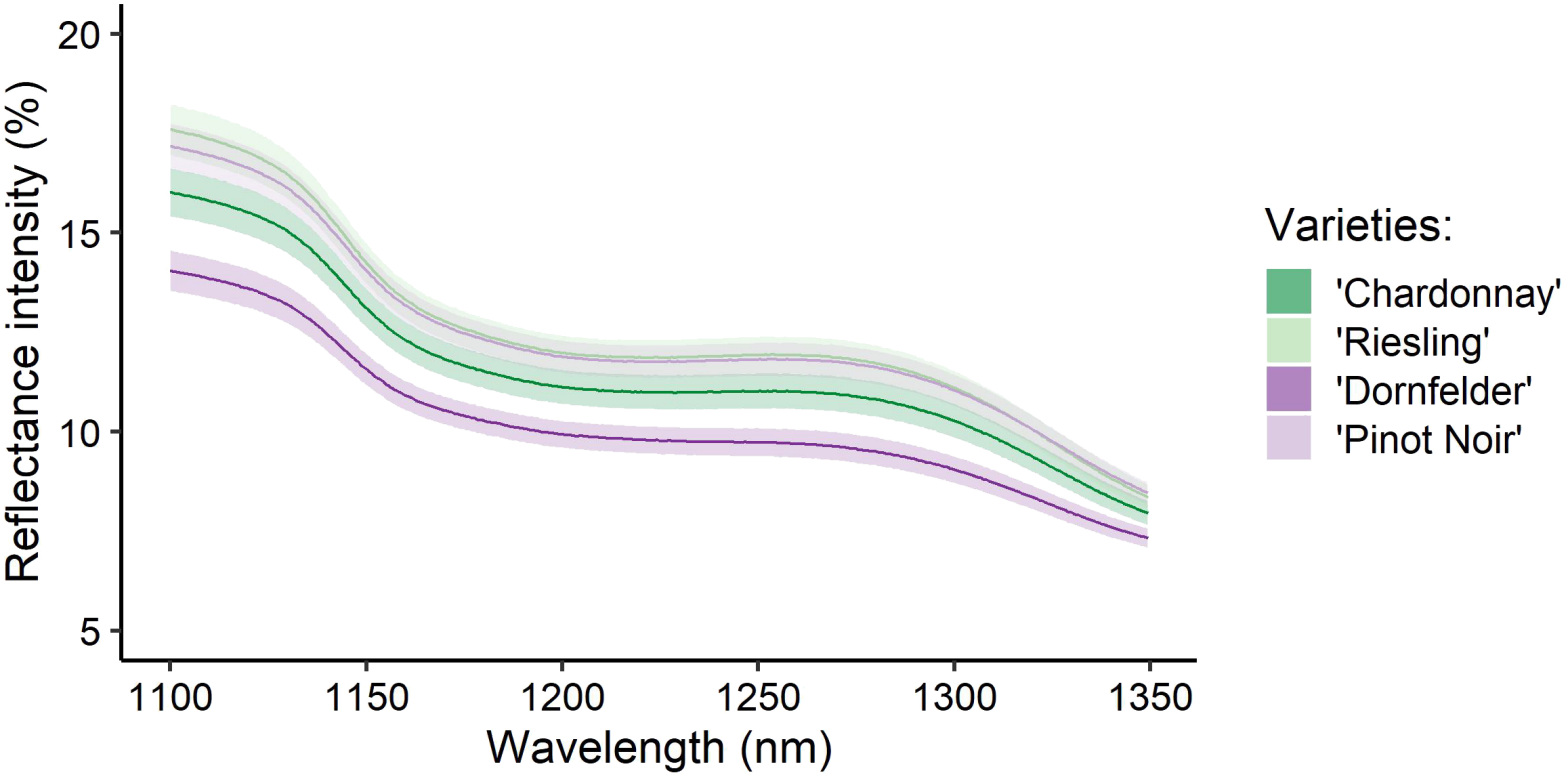
Averaged recorded spectra of berries from the *Vitis vinifera* (L.) varieties ‘Chardonnay’ (dark green), ‘Riesling’ (light green), ‘Dornfelder’ (dark purple), and ‘Pinot Noir’ (light purple) used to train the model and collected at the timepoints T1-T5 (see **Table 1**). Depicted are the recorded and referenced spectra without pre-treatments and their respective 95% confidence interval.

**Figure 5:**
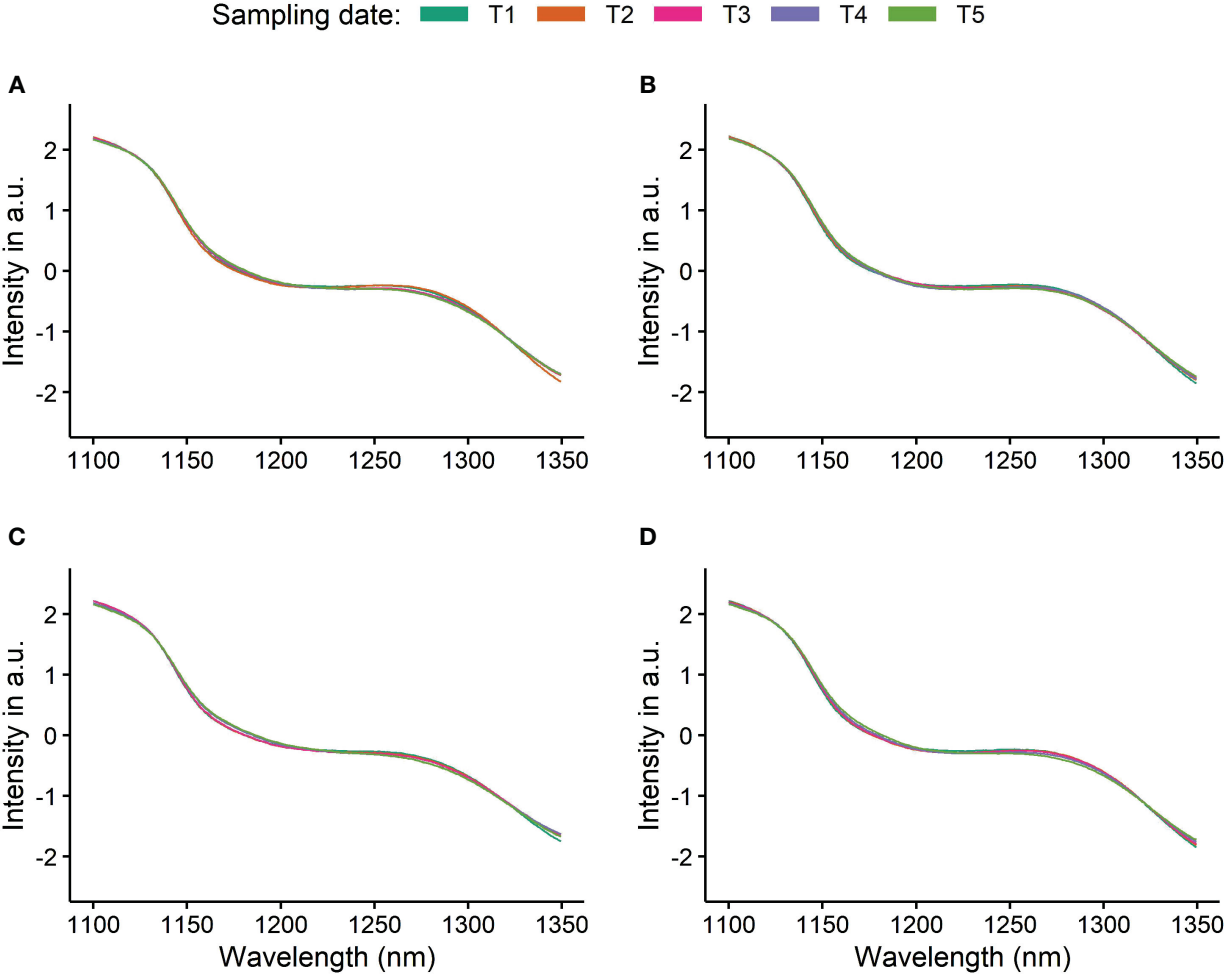
Pre-processed spectra of berries from the *Vitis vinifera* (L.) varieties ‘Chardonnay’ **(A)**, ‘Riesling’ **(B)**, ‘Dornfelder’ **(C)**, and ‘Pinot Noir’ **(D)** used to train a model to forecast fructose content over all four varieties and collected over all timepoints (T1-T5, see **Tables 1**–**3**). Depicted are the averaged, pre-treated (window size 5, polyorder 1) recorded and referenced spectra and their respective 95% confidence interval.

In total, 640 spectra and the corresponding reference values (see **Figure 3**) were gathered. However, two spectra of the white *Vitis vinifera*(L.) varieties (one of ‘Riesling’ and ‘Chardonnay’, respectively) were recognised as outliers and therefore had to be excluded. Exemplarily, spectra recorded from berries of the different *Vitis vinifera* (L.) varieties during ripening are presented in **Figures 4**, **5**. Obvious concerning the raw recorded spectra without pre-treatments are the differences in signal intensities between the varieties, with ‘Dornfelder’ showing lowest intensities. These differences were eliminated by SNV and spectra were smoothed by Savitzky-Golay filter (see **Table 3**) as can be seen in **Figure 5**. Differences between spectra of the different sampling time points remained low.

### 3.2 Reference values

The value ranges observed among the various varieties for the training data sets indicated a broad spectrum from unripe to ripe grapevine berries. Sugar contents ranged from 1.76g/L to 136.45g/L and 4.65g/L to 136.44g/L for fructose and glucose, respectively. Acid contents varied from 0.31g/L to 30.39g/L and 5.58g/L to 19.21g/L for malic acid and tartaric acid, respectively. Regarding individual varieties, the narrowest value range was noted for tartaric acid, displaying its highest concentrations in ‘Riesling’ (6.17g/L-19.21g/L) and lowest in ‘Pinot Noir’ (5.58g/L-15.09g/L) berries (see **Table 4**; **Figure 3**). For the other target substances fructose, glucose and malic acid, highest value ranges prevailed for the variety ‘Pinot Noir’, and lowest for ‘Dornfelder’.

**Table 4:** Value ranges and standard deviations (*σ*) in g/L of the target substances fructose, glucose, malic acid and tartaric acid for the different data sets for the training of the models.

	*Fructose*		*Glucose*		*Malic acid*		*Tartaric acid*	
	Value range (g/L)		Value range (g/L)		Value range (g/L)		Value range (g/L)	
	*σ* (g/L)		*σ* (g/L)		*σ* (g/L)		*σ* (g/L)	
*Model*	*R* ^²^	RMSE (%)	*R* ^²^	RMSE (%)	*R* ^²^	RMSE (%)	*R* ^²^	RMSE (%)
All	1.76–136.45 33.49		4.65–136.44 31.51		0.31–30.39 7.26		4.02–19.21 3.02	
	0.93	8.52 (6.32)	0.91	9.26 (7.03)	0.84	2.76 (9.18)	0.69	1.65 (10.89)
White	1.76–124.01 35.97		4.96–119.44 33.92		0.74–25.68 7.41		5.08–19.21 3.13	
	0.94	8.57 (7.01)	0.96	6.60 (5.77)	0.89	2.37 (9.52)	0.69	1.70 (12.05)
Red	2.51–136.45 30.64		4.65–136.44 28.70		0.31–30.39 7.06		4.02–15.09 2.41	
	0.92	8.18 (6.10)	0.89	9.16 (6.95)	0.82	2.72 (9.04)	0.71	1.24 (11.23)
CHA	2.82–124.01 36.77		7.87–119.44 33.74		0.74–25.68 7.69		5.08–14.90 2.47	
	0.96	7.25 (5.99)	1.00	1.47 (1.32)	0.90	2.33 (9.34)	0.76	1.18 (12.02)
RIE	1.76–113.94 34.47		4.96–118.68 33.31		1.04–23.65 7.13		6.17–19.21 3.07	
	0.94	8.37 (7.46)	0.92	8.84 (7.77)	0.91	1.99 (8.81)	0.76	1.44 (11.06)
DOR	3.04–101.65 25.22		9.31–107.36 23.96		0.47–22.55 5.94		4.02–14.22 2.30	
	0.95	5.36 (5.44)	0.95	5.23 (5.33)	0.94	1.35 (6.13)	0.82	0.93 (9.11)
PIN	2.51–136.45 35.19		4.65–136.44 32.86		0.31–30.39 7.93		5.58–15.09 2.15	
	0.97	5.78 (4.32)	0.89	10.30 (7.82)	0.85	2.82 (9.36)	0.69	1.14 (11.98)
Ø	0.94	7.43 (6.09)	0.93	7.27 (6.00)	0.88	2.32 (8.77)	0.73	1.33 (11.19)

Models were trained either using all collected spectra (All), spectra of a distinct berry colour (Red and White, respectively) or for one of the individual *Vitis vinifera* (L.) varieties, comprising ‘Chardonnay’ (CHA), ‘Riesling’ (RIE), ‘Dornfelder’ (DOR) and ‘Pinot Noir’ (PIN). Depicted are the corresponding coefficients of determination (R^²^) and root mean square errors (RMSE) in g/L, with the relative to the value ranges corresponding percentages in parentheses, per model and averaged (Ø) over all models for the respective target substances.

To avoid extrapolation, the Kennard Stone algorithm was selected to ensure an independent test set remaining within the boundaries of the training sets value range. These test sets exclusively comprised berries gathered from grapevines that have generally attained ripeness. This was typified by elevated sugar levels, ranging from 49.02g/L to 126.95g/L and from 49.80g/L to 125.19g/L for fructose and glucose, respectively, and relatively low acidity, ranging from 0.43g/L to 5.55g/L and from 4.31g/L to 11.73g/L for malic acid and tartaric acid, respectively (see **Table 5**). The observed value ranges and standard deviations (*σ*) in the test sets were highest for the variety ‘Chardonnay’ concerning sugars (49.02g/L-126.95g/L, *σ* = 19.19g/L for fructose; 49.80g/L-112.81g/L, *σ* = 18.26g/L for glucose) and for ‘Riesling’ regarding the two acids (0.43g/L-3.91g/L, *σ* = 0.91g/L for malic acid; 5.59g/L-9.61g/L, *σ* = 0.96g/L for tartaric acid). Smallest value range in the test set was again found for the variety ‘Dornfelder’ for all four target substances.

**Table 5:** Value ranges (min - max, in g/L) and standard deviations ( σ , in g/L) of the independent test sets for the target substances and the *Vitis vinifera* (L.) varieties ‘Chardonnay’, ‘Riesling’, ‘Dornfelder’ and ‘Pinot Noir’.

Analyte	‘Chardonnay’	‘Riesling’	‘Dornfelder’	‘Pinot Noir’
*Fructose*				
min-max	49.02–124.00	59.12–113.59	59.68–99.51	70.96–126.95
*σ*	19.19	12.93	8.80	13.76
All	7.56 (10.08)	10.00 (18.36)	7.54 (18.92)	8.87 (15.84)
Colour	7.63 (10.17)	10.08 (18.51)	8.18 (20.53)	8.09 (15.89)
Individual	8.74 (11.65)	8.19 (15.03)	7.61 (19.10)	7.94 (14.18)
Ø	7.98 (10.64)	9.42 (17.30)	7.78 (19.52)	8.57 (15.31)
*Glucose*				
min-max	49.80–112.81	57.05–105.39	65.90–100.47	70.42–125.19
*σ*	18.26	13.47	8.91	13.54
All	7.86 (12.47)	9.63 (19.92)	7.21 (20.84)	8.65 (15.79)
Colour	8.59 (13.64)	9.37 (19.39)	6.97 (20.16)	8.62 (15.73)
Individual	9.32 (14.79)	9.00 (18.61)	8.93 (25.84)	7.69 (14.03)
Ø	8.59 (13.63)	9.33 (19.31)	7.70 (22.28)	8.32 (15.18)
*Malic acid*				
min-max	0.77–4.37	1.29–5.55	0.57–3.24	0.43–3.91
*σ*	0.75	0.95	0.53	0.91
All	2.57 (71.31)	2.33 (54.62)	2.01 (75.17)	2.27 (65.15)
Colour	2.67 (74.03)	2.31 (54.20)	2.49 (93.38)	2.20 (63.14)
Individual	3.29 (91.50)	2.55 (59.73)	2.43 (90.87)	2.39 (68.69)
Ø	2.84 (78.95)	2.40 (56.18)	2.31 (86.47)	2.29 (65.66)
*Tartaric acid*				
min-max	5.14–8.72	6.33–11.73	4.31–7.24	5.59–9.61
*σ*	0.72	1.09	0.70	0.96
All	1.31 (36.71)	1.80 (33.24)	1.49 (50.79)	1.17 (28.93)
Colour	1.49 (41.81)	1.63 (30.19)	1.29 (44.07)	1.26 (31.37)
Individual	1.09 (30.61)	1.32 (24.41)	1.21 (41.41)	1.06 (26.28)
Ø	1.30 (36.38)	1.58 (29.28)	1.33 (45.42)	1.16 (28.86)

The calculated models using all gathered spectra (All), spectra of the respective colour of the berries (Colour) or spectra of the individual variety (Individual) were applied on the test sets and root mean square errors of prediction (RMSEP) and the respective mean (Ø) are depicted in g/L with the relative to the value ranges corresponding percentages in parentheses: RMSEP (%).

In order to be able to estimate the average maturity of the whole vineyards, conventionally used hundred-berries-samples were taken evenly from the plots (see **Figure 1**). Must was extracted and the levels of the target analytes were measured using HPLC. Utilising the 60 berries from the independent test sets, it was possible to approximate these values by averaging the mean and median of the dataset. The mean and median values may either underestimate or overestimate the contents, and this method automatically facilitates selecting the closest or second closest value, as illustrated in **Table 6**, particularly for sugar contents.

**Table 6:** True contents of quality determining substances at harvest for the vineyards of the *Vitis vinifera* (L.) varieties ‘Chardonnay’ (CHA), ‘Riesling’ (RIE), ‘Dornfelder’ (DOR) and ‘Pinot ‘Noir’ (PIN) measured with high-performance liquid-chromatography, estimated using one hundred-berries-samples (MS) and the mean (m), the median(md) contents of 60 berries and the respective average of both (Ø_m md_).

Analyte		CHA	RIE	DOR	PIN	Ø_deviation from MS_
*Fructose*	MS	95.55	85.35	82.33	103.69	
	m test	92.64	84.61	81.62	100.73	1.83
	md test	98.35	85.16	82.45	103.14	0.92
	Ø_m md_	95.50	84.89	82.04	101.94	0.64
*Glucose*	MS	92.05	86.15	86.26	100.87	
	m test	89.39	85.00	85.38	98.38	1.80
	md test	96.06	85.35	85.17	98.57	2.05
	Ø_m md_	92.73	85.18	85.28	98.48	1.26
*Malic acid*	MS	2.03	2.68	2.19	2.27	
	m test	2.14	2.76	2.16	2.13	0.09
	md test	2.04	2.52	2.16	1.96	0.13
	Ø_m md_	2.09	2.64	2.16	2.05	0.09
*Tartaric acid*	MS	7.24	9.49	5.35	8.35	
	m test	7.06	8.98	5.55	7.36	0.47
	md test	7.17	8.88	5.45	7.40	0.43
	Ø_m md_	7.12	8.93	5.50	7.38	0.45

All analytes are measured and depicted in g/L.

Evaluating average ripeness using the prediction of the test data set (see **Supplementary Material, Tables S2**, **S3**) a comparable picture emerges, as the prediction for the individual berries tend to either underestimate or overestimate the contents. Calculating the average maturity using the same approach, revealed good results. Average fructose and glucose contents could be predicted with a deviation from the estimation using the true reference values ranging from 0.40g/L to 6.35g/L per sugar. The deviation for the prediction of malic acid and tartaric acid contents ranged from 0.02g/L to 1.08g/L and from 0.07g/L to 1.17g/L, respectively.

### 3.3 Statistical evaluation and modelling

Using this spectral dataset, different kind of models were trained using either the spectra of berries from all varieties (here referred to as *all*), from varieties with the same berry colour (here referred to as *colour*) or by using spectra of berries from the individual varieties (here referred to as *individual*). The coefficients of determination (*R* ^2^) of the training, as well as the RMSEP of the tests using an independent test set of each variety are shown in **Tables 4**, **5**. As Minasny and Mcbratney (2013) already showed, RPD values increase as *R* ^2^ increases. To keep a clear presentation of the results in **Table 4** the RPD values are accessible in **Supplementary Material Table S1**.

The *R* ^2^ values of the training sets and RMSEP of the test sets, as depicted in **Tables 4**, **5**, indicate a reliable forecasting for the two sugars and malic acid across all *Vitis vinifera* (L.) varieties using the different models. Specifically, coefficients of determination ranged from 0.89 to 1.0 for the sugars and 0.82 to 0.94 for malic acid, respectively. The *R* ^2^ values for tartaric acid predictions ranged from 0.69 to 0.82, with the best results being achieved with models for the individual varieties, also reflected in RMSEP. Averaged coefficients are in included in **Table 4**.

**Figure 6:**
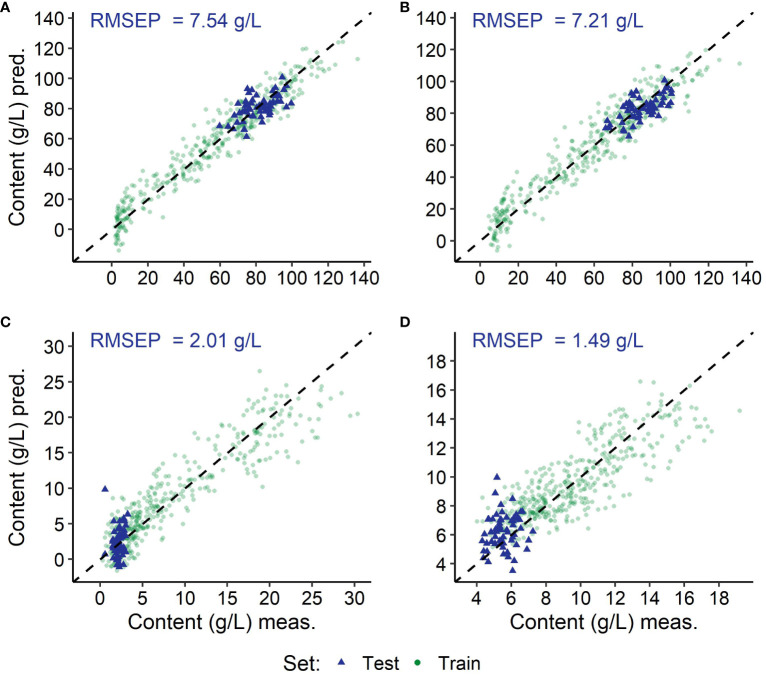
Results of the predictions using the modelling with spectra of all *Vitis vinifera* (L.) varieties; depicted are the training (green) and the independent test (blue) datasets of the grapevine variety ‘Dornfelder’ per target substance: fructose **(A)**, glucose **(B)**, malicacid **(C)** and tartaric acid **(D)**, as well as the root mean square error of prediction from the independent test set (RMSEP). Coefficient of determination (*R* ^2^) and root mean square error (RMSE) of the modelling can be found in **Table 4** and values of the residual prediction derivation of the training in **Supplementary Table S1**.

**Figure 7:**
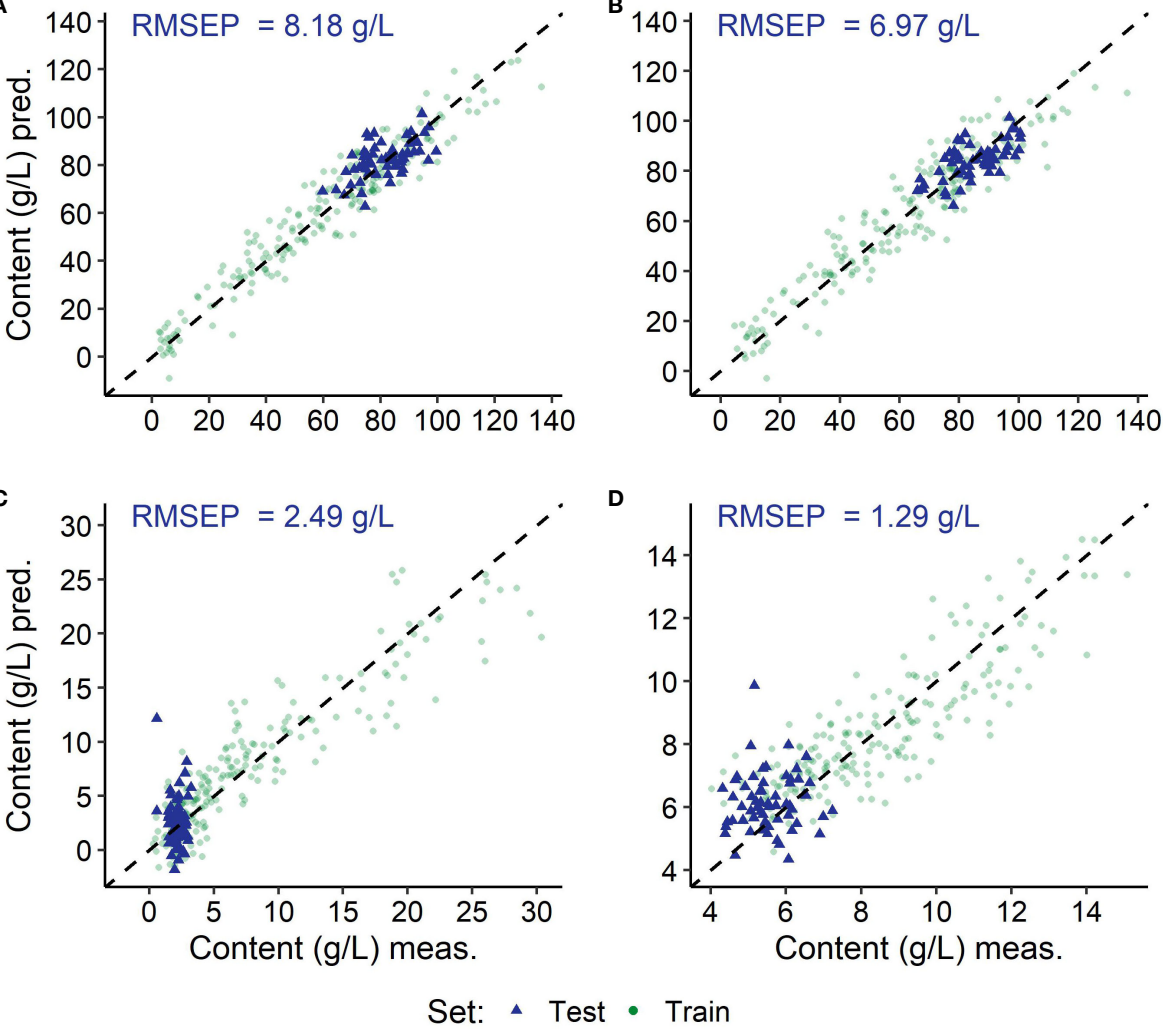
Results of the predictions using the modelling with spectra of *Vitis vinifera* (L.) varieties with red berries (‘Dornfelder’ and ‘Pinot Noir’); depicted are the training (green) and the independent test (blue) data sets of the grapevine variety ‘Dornfelder’ per target substance: fructose **(A)**, glucose **(B)**, malic acid **(C)** and tartaric acid **(D)**, as well as the root mean square error of prediction from the independent test set (RMSEP). Coefficient of determination (*R* ^2^) and root mean square error (RMSE) of the modelling can be found in **Table 4** and values of the residual prediction derivation of the training in **Supplementary Table S1**.

**Figure 8:**
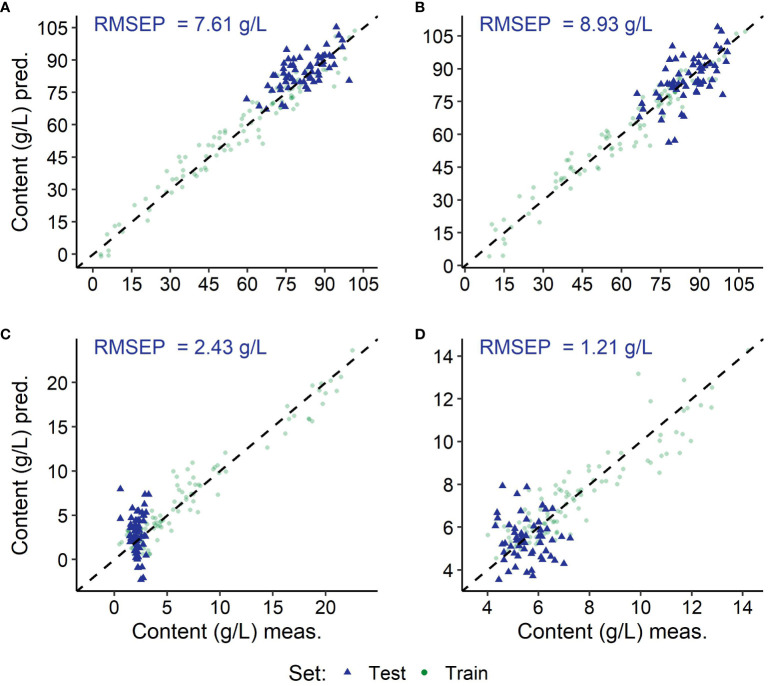
Results of the predictions using the modelling with spectra of the berries from the *Vitis vinifera* (L.) variety ‘Dornfelder’; depicted are the training (green) and the independent test (blue) data sets per target substance: fructose **(A)**, glucose **(B)**, malic acid **(C)** and tartaric acid **(D)**, as well as the root mean square error of prediction from the independent test set (RMSEP). Coefficient of determination (*R* ^2^) and root mean square error (RMSE) of the modelling can be found in **Table 4** and values of the residual prediction derivation of the training in **Supplementary Table S1**.

The training set gaining lowest absolute RMSEs (see **Table 5**) was the test set of the *Vitis vinifera* (L.) variety ‘Dornfelder’, exemplarily depicted in **Figures 6**–**8**. Test sets, depicted in blue, remain in the value range of the training sets. In relation to the test sets value range, the variety ‘Chardonnay’ showed best results for the prediction of both sugars, and ‘Riesling’ and ‘Pinot Noir’ for the acids malic acid and tartaric acid, respectively.

The attribution map in **Figure 2** reveals differences between the spectral ranges crucial for predicting the target substances, especially comparing sugars and acids. For sugars key spectral bands are situated between 1100nm-1120nm and 1130nm-1145nm. In contrast, the impacts of bands around 1230nm and between 1320nm-1325nm remain minimal. Concerning acids, spectral bands between 1100nm and 1150nm are significant too. However, for predicting malic acid, seven peaks were identified, with the highest at 1120nm-1125nm. This band is not crucial for predicting sugars, as well as the influence of the region above 1340nm. In the case of acids, the region above 1300nm has a more pronounced impact on the RMSEP compared to sugars. Despite low influences through low prediction capabilities, in contrast to malic acid, tartaric acid seems to show a superposition of two peaks at 1120nm-1125nm.

## 4 Discussion

The findings presented in this study broaden the perspective on predicting maturity- and quality-determining components in wine grapes. A spectrometer non-destructively capturing spectral data within the range of 1100nm to 1350nm was employed for predicting the levels of individual sugars and acids in berries of four *Vitis vinifera* (L.) grapevine varieties. A large data set was generated, with the help of which various, automatically optimised models could be built to predict the levels of the target substances in the respective varieties. Promising results could be achieved using the differently calculated models for the prediction of both sugars and malic acid.

With this study, previous findings can be confirmed, as the modelling showed high average prediction accuracies for the sugars with coefficients of determination over 90% and low RMSEs (see **Table 4**) (Donis-González et al., 2020; Ferrara et al., 2022a, 2022). In comparison to the mentioned investigations, using table grapes, wine grapes have other ripeness criteria concerning their constituents. They are richer in polyphenols, especially in red varieties (Kok, 2017), and show higher sugar and acid contents (Liu et al., 2006a), essential for the production of high-quality wines. Nonetheless, in comparison to wine grapes, table grapes are typically larger, feature thinner skins and, in some cases, lack seeds. It is important to bear this in mind when making comparisons with earlier studies, as the depth of light penetration into the grapevine berry, the impact of skin thickness, and the presence of seeds have not been conclusively explored until now (Lammertyn et al., 2000; Fraser et al., 2001; Nicolaï et al., 2007; Pratiwi et al., 2023). In their study, Jenne and Zappe (2023) demonstrated that the skin of seedless table grapes exhibits a greater.

Scattering coefficient and more forward scattering compared to the flesh, possibly due to a higher density of cells. This implies that an increased thickness of the berry skin could possibly amplify these effects.

However, in contrast to most of the earlier studies (Nicolaï et al., 2007; dos Santos et al., 2013) using soluble solids content (TSS or SSC in °Brix) and titrable acid (TA in g malic acid/L) or pH, this analysis predicts individual sugars and acids. This provides significant value for the wine maker, as it enables for example the calculation of the glucose-to-fructose ratio, where a ratio near 1 indicates ripe berries for *Vitis vinifera* (L.) varieties (Kuhn et al., 2014). Conversely, continuous monitoring, specifically of malic acid contents, can help ensuring their sufficiently high contents. This is especially crucial as rising temperatures (Rienth et al., 2016) and severe water scarcity (Shi et al., 2023) can enhance malic acid respiration.

Comparing the four *Vitis vinifera* (L.) varieties used for this study, there are noticeable differences in berry sizes between the varieties. Ferrara et al. (2022a) and Ferrara et al. (2022b) investigated the prediction of sugar contents using a NIR sensor and achieved better results with table grapes than with wine berries. Nevertheless, they suspect that berry size might be a factor too. Observations in the years 2020 and 2021 revealed that ripe ‘Dornfelder’ grapevine berries exhibit by far the largest size with up to 3.88cm^3^/4.04cm^3^.

This is followed by ‘Chardonnay’ (1.41cm^3^/1.47cm^3^) and ‘Pinot Noir’ (1.38cm^3^/1.51cm^3^), which are more similar in this regard (see **Supplementary Table S4**), and ‘Riesling’ berries were smallest (1.24cm^3^/1.27cm^3^). A comparable pattern can be observed in the intensities of the recorded spectra, depicted in **Figure 4**, with ‘Dornfelder’ berries showing the lowest intensities in unprocessed spectra. A minor portion of the light enters the fruit and is reflected back to the detector, conveying data about the ingredients. However, most of the light comes from the background, which accounts for the obvious differences in the unprocessed spectra. According to Mejía-Correal et al. (2023), there were no obvious differences between spectra of red and white grapevine varieties in the observed wavelength range. Due to low intensities, spectral changes between sampling dates are weak too. Also striking is that the value ranges of the training and the test data sets for ‘Dornfelder’ are the smallest, except for the training data concerning the tartaric acid. Nevertheless, prediction accuracies for this variety remained high with a *R* ^2^ of 95% for both sugars, 94% for malic acid and 82% for tartaric acid (**Table 4**). For the independent test (**Table 5**) and the two sugars, fructose and glucose, RMSEP were lowest with on average 7.78 g/L and 7.70g/L, respectively, and low for the malic acid with a RMSEP of 2.31 g/L too. Contrary to this, RMSEP of ‘Riesling’ berries were highest concerning sugars (8.19 g/L - 10.08 g/L), despite moderate value ranges and *R* ^2^ values of the training ranging from 76% to 94%. Using spectra recorded in transmission mode and gaining higher intensities, ‘Riesling’ and ‘Dornfelder’ showed best predictions (Cornehl et al., 2023) for the sugars. There, the RMSEP of the validation set for ‘Riesling’ and ‘Dornfelder’ were 5.16g/L and 5.17g/L, and from 5.26g/L and 4.09g/L, for fructose and glucose, respectively. The portion of reflected light in the measured intensity in reflectance mode is low and a substantial part of the intensity stems from the background, thereby reducing the informational value (Lammertyn et al., 2000). This would be particularly the case with very small berries, or if the light entry surface diameter of the sensor is large. Exploring the underlying reasons for these differences and the influences on the modelling and prediction accuracies is essential to further enhance precision for a future use in viticulture.

Comparing the different modellings, it is noticeable that ‘Chardonnay’ and ‘Dornfelder’ predictions showed lowest RMSEP using the “All” model, concerning the best predictable target substances fructose, glucose and malic acid, as can be seen in **Table 5**. Despite the obvious over-fitting of the model for glucose for the ‘Chardonnay’ berries in the individual model (RPD=21.74 in **Supplementary Table S1**, *R* ^2^ = 1.00, RMSE = 1.47g/L in **Table 4**), RMSEP of the test set was not excessively high with 9.32g/L. In comparison, the other varieties showed RMSEP of 9.00g/L, 8.93g/L and 7.69g/L for ‘Riesling’, ‘Dornfelder’ and ‘Pinot Noir’, respectively (**Table 5**). This overfitting could have been prevented by reducing the components and would possibly lower the tests RMSEP. Due to the brute-force approach, this was not done, to provide a clear view on the results by automatic optimisation using this technique. By narrowly limiting the variables to be tested, the calculation time and required computing capacity could also be reduced.

Regarding RMSEP for the test sets, it is noticeable that the influences of different modelings are relatively low for sugar contents, with the largest observed in the modelings for fructose and ‘Riesling’ berries (see **Table 5**). However, these predictions are associated with the forecasts for individual berries that comprise the test sets. An initial promising attempt was made to estimate the average contents of quality-determining substances using this dataset, as the intention is to utilise this sensor for average ripening prediction in viticulture. Following Kasimatis and Vilas (1985) findings, it was concluded that the test set was adequate, comprising 60 individual berries per variety. Increasing the sample size would result in a marginal improvement of accuracy. This can be proven with **Table 6**, as mean, median and the average of both showed low differences compared to the respective hundred-berries-samples concerning sugars (± 0.64 g/L- ± 2.05 g/L) and acids (± 0.09g/L- ± 0.47 g/L).

The performance of the models (*R* ^2^) has a significant impact on the predicted estimations of the average ripeness compared to the true estimations. Especially if the model is not overfitted, which typically results in high prediction errors. Although this happened for glucose prediction in ‘Chardonnay’, the estimation of the average ripeness remained reliable. It is challenging to predict tartaric acids contents (Cornehl et al., 2023), as they showed low *R* ^2^ values, even with spectra recorded in transmission mode and higher intensities. Here, the estimations using models for tartaric acid showed comparable differences between estimated true and estimated predicted average contents like malic acid. As can be seen in **Supplementary Tables S2**, **S3** in **the Supplementary Material**, the deviations for both acids are comparable estimating their average amount, even though the coefficients of determination of the training sets for tartaric acid (*R* ^2^ = 0.69–0.82) were lower compared to malic acid (*R* ^2^ = 0.82–0.94).

As mentioned by Walsh et al. (2020) for sorting purposes, a technique makes a valuable contribution if the standard deviation is greater than the error of the prediction. The observed standard deviations in the independent test sets ranged from 8.80 g/L to 19.90 g/L and from 0.53 g/L to 1.09 g/L for both sugars and acids, respectively (**Table 5**). There it can be noted, that this applies only to the sugars and the RMSEP concerning individual berries. However, average contents in the 60 individual berries were predicted using the NIRONE Sensor S1.4 sensor with deviations from the estimated average contents between 0.40 g/L and 6.25 g/L for the sugars, and 0.02 g/L and 1.17 g/L for the acids (**Supplementary Tables S2**, **S3**). Comparing these with the standard deviations of the test set in **Table 5**, it can be seen, that for sugars, again all observed RMSEP are lower than the observed standard deviations. Based on this evidence, the most precise predictions were achieved for both carbohydrates, especially notable for ‘Chardonnay’ in terms of fructose content. The acids could also be reasonably estimated, demonstrating notable prediction accuracy, particularly for malic acid in ‘Dornfelder’. In this case, the standard deviation was 0.53 g/L, while the prediction differences were 0.02g/L and 0.14g/L using the models with all spectra and spectra of the red berries, respectively. Only the prediction of the average malic acid contents for the variety ‘Riesling’ was not accurate enough, with any of the models created, and for the variety ‘Dornfelder’ using the individual modelling. For tartaric acid prediction, using the model calculated across all *Vitis vinifera* (L.) varieties, the difference between estimated and predicted average contents was greater than the variances for the test sets of ‘Riesling’ and ‘Dornfelder’. However, average tartaric acid prediction was best using the individual modelling with low differences from 0.07g/L to 0.08g/L and a standard deviation ranging from 0.70g/L to 1.09g/L.

For the practical use, the correct estimation of the average ripeness of the vineyard is decisive, as the winegrowers are often paid by cooperatives on the basis of the contained sugars. In literature an error of 10 g/L (or 1° Brix) for sugars and 0.5 g/L for acids are told to be acceptable (Mejean Perrot et al., 2022). In this study we were able to show that two varieties benefited from the mixed models, while for the other two the calibration based on the individual variety worked best (see Tables **Supplementary Tables S2**, **S3**). Nonetheless, when summing up the main stored sugars, fructose and glucose, the average ripeness predictions using this sensor and an independent test set revealed high accuracies. The differences between the predictions and the true estimations remained in all models and for all varieties under 10g/L, except for Riesling and the “All”-model with 10.27 g/L. Consequently, calibration is possible incorporating berries from other *Vitis vinifera* (L.) varieties in the calibration and an automatic optimisation process. This suggests practical applicability as there is most likely no need to create calibration models for hundreds of varieties. Moreover, the calibration models can be more readily tailored to the specific application based on the use case, for example with regard to the geographical region in which the measurement is being carried out. As Cozzolino et al. (2006) also noted, NIR spectroscopy solutions are applied mainly with costly scientific equipment in a destructive way, using homogenised samples. When using a non-destructive sensor, individual grapes must be measured, although the prediction for each berry is less critical than the overall assessment of the average ripeness.

This study aims to help surmount the existing obstacles to the commercial use of non-destructive near- infrared spectroscopy in viticulture. A potential future scenario could involve using sensors embedded in smartphones. Given the nearly universal connectivity to mobile data networks in many countries, a cloud-based evaluation of the collected data can be initiated. By dynamically creating calibrations in a cloud and the finding, that ingredient predictions can span different grapevine varieties, the costs of calibration and the associated application are minimised. Furthermore, guidelines for sample collection should be established and implemented. Future studies should also investigate how accurately average maturity can be predicted using such models in varieties not included in the calibration, how large spatial influences can affect such predictions, and the optimal number of individual berries required for this analysis. In addition, transformations should be integrated into the dynamic calibration process, enabling a transfer to other sensors in order to be able to follow the rapid technical progress, to minimise ongoing costs and thus to increase the acceptance of this technology by viticulturists.

## Data availability statement

The raw data supporting the conclusions of this article will be made available by the corresponding authors, without undue reservation.

## Author contributions

LC: Conceptualization, Formal analysis, Investigation, Methodology, Resources, Validation, Visualization, Writing – original draft, Writing – review & editing. PG: Formal analysis, Methodology, Software, Validation, Visualization, Writing – review & editing. XZ: Investigation, Methodology, Resources, Validation, Writing – review & editing. JK: Methodology, Software, Writing – review & editing. FS: Funding acquisition, Project administration, Resources, Writing – review & editing. RT: Funding acquisition, Resources, Writing – review & editing. RG: Funding acquisition, Project administration, Resources, Writing – review & editing. AK: Funding acquisition, Project administration, Resources, Writing – review & editing.
