# Supplementary material for: Non-destructive quantification of key quality characteristics in individual grapevine berries using near-infrared spectroscopy
Source: Front Plant Sci. 2024 Jul 5;15:1386951. doi: 10.3389/fpls.2024.1386951 (PMC11257901; doi:10.3389/fpls.2024.1386951)
Supplement: Supplementary file 1 [file DataSheet_1.pdf]

Supplementary Material

1 SUPPLEMENTARY TABLES AND FIGURES

Model		Fructose	Glucose	Malic acid	Tartaric acid
All		3.82	3.30	2.50	1.79
Col	white	4.10	5.00	3.00	1.80
	red	3.61	3.02	2.39	1.88
Ind	CHA	4.88	21.74	3.13	2.02
	RIE	3.97	3.65	3.40	2.06
	DOR	4.52	4.44	4.05	2.37
	PIN	5.85	3.06	2.58	1.81

Table S1. Values of the residual prediction derivation (RPD) of the training sets from different models. Models for the target substances (fructose, glucose, malic acid and tartaric acid) were calculated using spectra of white ('Chardonnay': CHA, 'Riesling': RIE), and red ('Dornfelder': DOR, 'Pinot Noir': PIN) *Vitis vinifera* (L.) varieties. Models for the prediction of the substances were generated with different spectral data subsets: spectra from berries of all varieties (*All*), from berries of a specific colour (*Col*; red and white) or from the individual varieties (*Ind*) were used.

Analyte	'Chardonnay'			'Riesling'		
Model:	*All*	*Col*	*Ind*	*All*	*Col*	*Ind*
Fructose		*95.50 g/L*			*84.89 g/L*	
mean	93.03	92.44	96.81	91.04	90.98	86.64
median	97.10	96.32	100.95	91.23	91.22	87.10
Ø	95.07	94.38	98.88	91.14	91.10	86.87
Diff.	0.44	1.12	3.38	6.25	6.21	1.98
Glucose		*92.73 g/L*			*85.18 g/L*	
mean	92.14	87.67	87.40	89.49	87.01	85.42
median	97.73	90.78	89.26	89.31	87.42	85.74
Ø	94.94	89.23	88.33	89.40	87.22	85.58
Diff.	2.21	3.51	4.40	4.22	2.04	0.40
Malic acid		*2.09 g/L*			*2.64 g/L*	
mean	2.21	2.95	3.2	3.66	3.73	3.66
median	2.08	2.21	2.34	3.57	3.7	3.58
Ø	2.15	2.58	2.77	3.62	3.72	3.62
Diff.	0.06	0.49	0.68	0.98	1.08	0.98
Tartaric acid		*7.12 g/L*			*8.93 g/L*	
mean	7.61	7.92	7.24	7.74	7.95	8.90
median	7.42	7.49	6.85	7.79	7.89	8.82
Ø	7.52	7.71	7.05	7.77	7.92	8.86
Diff.	0.40	0.59	0.08	1.17	1.01	0.07

Table S2. Mean and median predicted contents of quality determining substances at harvest for the vineyards of the white *Vitis vinifera* (L.) varieties 'Chardonnay' and 'Riesling' using three different models (all, colour, individual) as shown in Table 2. Depicted are the estimated true contents of the target analytes (in italics) as depicted in Table 6, as well as mean and median of the predicted contents of the independent test set comprising 60 berries, the average of both (Ø), and the respective absolute difference (Diff.) to the estimated true contents. All analytes are measured, predicted and depicted in g/L.

Analyte	'Dornfelder'			'Pinot Noir'		
Model:	*All*	*Col*	*Ind*	*All*	*Col*	*Ind*
Fructose		*82.04 g/L*			*101.94 g/L*	
mean	81.23	82.35	84.76	105.58	105.46	104.29
median	81.83	82.82	84.44	104.89	105.11	104.99
Ø	81.53	82.585	84.6	105.24	105.29	104.64
Diff.	0.51	0.54	2.56	3.30	3.35	2.70
Glucose		*85.28 g/L*			*98.48 g/L*	
mean	83.02	84.11	85.06	103.93	103.77	102.06
median	83.26	84.50	83.97	105.73	105.30	103.69
Ø	83.14	84.31	84.52	104.83	104.54	102.88
Diff.	2.14	0.97	0.77	6.35	6.05	4.40
Malic acid		*2.16 g/L*			*2.05 g/L*	
mean	2.35	2.56	2.78	1.48	1.77	2.00
median	1.94	2.03	2.65	1.1	1.14	1.53
Ø	2.15	2.295	2.715	1.29	1.46	1.77
Diff.	0.02	0.14	0.56	0.76	0.60	0.29
Tartaric acid		*5.50 g/L*			*7.38 g/L*	
mean	6.23	6.17	5.45	6.96	6.68	7.34
median	6.23	6.05	5.4	6.9	6.59	7.28
Ø	6.23	6.11	5.43	6.93	6.64	7.31
Diff.	0.73	0.61	0.07	0.45	0.75	0.07

Table S3. Mean and median predicted contents of quality determining substances at harvest for the vineyards of the red *Vitis vinifera* (L.) varieties 'Dornfelder' and 'Pinot Noir' using three different models (all, colour, individual) as shown in Table 2. Depicted are the estimated true contents of the target analytes as depicted in Table 6, as well as mean and median of the predicted contents of the independent test set comprising 60 berries, the average of both (Ø), and the respective absolute difference (Diff.) to the estimated true contents. All analytes are measured, predicted and depicted in g/L.

	CHA	RIE	DOR	PIN
2020				
min	0.76	0.61	1.01	0.83
mean	1.41	1.27	2.12	1.51
median	1.41	1.25	2.05	1.45
max	2.10	1.89	3.88	2.41
σ	0.31	0.27	0.60	0.31
2021				
min	0.62	0.78	1.15	0.80
mean	1.47	1.24	2.23	1.38
median	1.42	1.24	2.23	1.33
max	2.47	2.19	4.04	2.16
σ	0.33	0.25	0.67	0.36

Table S4. Volume in cm^3 of individual grapevine berries from the *Vitis vinifera* (L.) varieties 'Chardonnay' (CHA), 'Riesling' (RIE), 'Dornfelder' (DOR) and 'Pinot Noir' (PIN), collected between Septembre 20th and 24th in 2020 and 2021. Vineyards were located in Rhineland palatinate at "Wollmesheimer Mütterle". Berries were collected from four plots per variety, including the one used for the presented study, and comprised in total 64 individual berries of each variety in the corresponding year. Depicted are the minimum (min), mean, median, maximum (max) volume, and the respective standard deviation (σ). Volume was calculated using the formula for an ellipsoid and the horizontal and vertical diameter of the berries, measured with a caliper.
